# Changes of factors associated with vaccine hesitancy in Chinese residents: A qualitative study

**DOI:** 10.3389/fpubh.2022.929407

**Published:** 2022-09-20

**Authors:** Sigui Long, Jingying Wu, Shile Wang, Yaqi Zhao, Jianli Wang, Shuangyu Zhao, Qing Niu, Hui Jin

**Affiliations:** ^1^Department of Epidemiology and Health Statistics, School of Public Health, Southeast University, Nanjing, China; ^2^Key Laboratory of Environmental Medicine Engineering, Ministry of Education, School of Public Health, Southeast University, Nanjing, China

**Keywords:** vaccine hesitancy, qualitative study, China, influential factor, return visit

## Abstract

**Introduction:**

There is an urgent need to address vaccine hesitancy to achieve booster vaccination. This study aimed to reveal the factors associated with vaccine hesitancy (including COVID-19 vaccine) among Chinese residents, address modifications of the factors since the previous year, and propose vaccination rate improvement measures.

**Materials and methods:**

This qualitative return visit study was performed between January and mid-February 2022, following the last interview conducted between February and March 2021. According to an outline designed in advance, 60 Chinese residents from 12 provinces participated in semi-structured interviews.

**Results:**

Vaccine safety was the biggest concern raised by respondents, followed by self-immunity and vaccine effectiveness, eliciting concern since the interview last year. Notably, online media accounted for a more significant portion of suggestion sources than before, and fear of pain was a novel factor affecting vaccine hesitancy. Moreover, unlike other areas, those from provinces with a per capita gross domestic product of 3–5 (RMB 10,000) reported less concern about vaccine price and effectiveness. They tended to seek advice *via* online media less and were greatly influenced by vaccination policies.

**Conclusions:**

Influential factors of vaccine hesitancy among Chinese residents are changing dynamically. Monitoring these trends is essential for public health measures and higher vaccination levels.

## Introduction

The global confirmed coronavirus disease (COVID-19) cases were still on the rise, and as of 18 February 2022, they had reached 419.0 million (1). On the same day, 137 new confirmed cases in 31 provinces were reported in China (2), and the fifth wave of COVID-19 in Hong Kong has drawn extensive attention. In the last 2 years, travel bans, mask-wearing, isolation, and nucleic acid testing have been rigorously implemented to respond to the pandemic. Unfortunately, the rapid spread of Delta and Omicron (3) has become a severe obstacle to ending the pandemic. Chronic prevention and control measures are insufficient to curb this pandemic. Vaccination effectively suppresses pandemics, alleviates their socioeconomic impact, and is an established strategy to prevent infectious diseases (4).

A recent study found that taking two doses of the BNT162b2 vaccine had 93.7% and 88.0% efficacy against alpha and delta variants, respectively (5). The Omicron variant has become the dominant epidemic strain globally. The neutralization efficiency against the Omicron variant was enhanced 100 times after receiving the third dose of the BNT162b2 vaccine (6). Receiving the third mRNA-1273 vaccination enabled us to detect neutralizing titres against the Omicron variant in all participants six months later (7). In addition, receiving a heterologous boost of adenovirus-vectored vaccine (AdV) after receiving two doses of inactivated vaccines (IAV) induced neutralizing antibody levels five times higher than a homologous boost (8). The above results suggest that a vaccine booster is vital for superior protection and reduces the risk of variant infection.

The absence of devastation caused by vaccine-preventable diseases and the spread of anti-vaccine movements through social media (9) undermine the role of vaccines in defending against infectious diseases. Being hesitant about receiving a vaccination or refusing vaccination when one is capable of doing so, namely “vaccine hesitancy,” was on the list of the top 10 threats to global health (10).

An online survey (11), carried out from January to March 2021 in seven cities in China, estimated the rate of COVID-19 vaccine hesitancy to be approximately 15.6%. The student group ranked first (23.9%). Those who received negative information about the COVID-19 vaccine or questioned the source of information were more likely to delay vaccination. In the subsequent 4 months, another investigation illustrated that 56.4% of diabetes patients in two hospitals had COVID-19 vaccine hesitancy, resulting from safety concerns and opinion discrepancies with doctors (12). In mid-August 2021, 22.2% of healthcare students in northwestern China showed unwillingness to COVID-19 vaccination due to vaccine safety and effectiveness (13). Another online study in the same month discovered that the rate of COVID-19 vaccine hesitancy among Chinese adults aged 18 years or older was modest (~ 8.4 %). Vaccination is promoted by lower vaccine conspiracy beliefs, more convenient vaccination services, and more trust in doctors and vaccine developers (14). These findings showed a downward trend in collective COVID-19 vaccine hesitancy among Chinese residents, but the hesitancy of specific groups (e.g., people with other health problems and students) was higher. The overall inoculation rate in China exceeded 85% (15). Therefore, it is imperative to understand and address vaccine hesitancy to bring COVID-19 under control and return to the world without severe acute respiratory syndrome coronavirus (SARS-CoV-2).

Researchers conduct qualitative studies by observing or interacting with people to collect data relevant to the phenomenon of interest. Last spring, we conducted in-depth interviews with Chinese residents to identify the factors influencing vaccine hesitancy. The study demonstrated that vaccine safety, price, effectiveness, and acquisition of professional suggestions were responsible for the reluctance to vaccination (16). Given that vaccine hesitancy is complicated and sets a specific phenomenon, differing in time, place, policies, and vaccines (17, 18), we paid a return visit to capture their perceptions and attitudes toward vaccination the following year, within the context of variant ravaging and vaccine booster popularization. The two interview results were compared to determine the factors influencing vaccine hesitancy in China. Dynamic monitoring of vaccine hesitancy is crucial for identifying unsolved barriers to herd immunity and novel factors affecting vaccination decisions. This study sought to elucidate the factors hindering vaccine uptake, address their modifications since the previous year, and provide policymakers with reference in facilitating booster vaccination to contain the COVID-19 pandemic.

## Materials and methods

### Study design

This qualitative study was conducted using an individual in-depth interview about vaccine hesitancy. Interviewers primarily used open-ended questions to avoid influencing the participant's opinions and were required to interact with interviewees based on the interview framework. Researchers guided and controlled the interview content appropriately to prevent the interviewee from expressing bias.

The semi-structured interview framework consisted of three components: (1) general information— mainly comprising the participants' age, gender, occupation, annual family income, and education level; (2) 12 open-ended questions about self-funded vaccine hesitancy, based on health beliefs and planned behavior theory, including personal knowledge and attitude toward vaccines, other people's impact, and other factors influencing vaccination; and (3) four types of open-ended questions enquiring about COVID-19 vaccines: (a) how well people understand information about COVID-19 vaccines; (b) how they get access to information about COVID-19 vaccines; (c) how they get to know COVID-19 vaccines; and (d) how their lifestyles have changed since the pandemic outbreak. Section one and two were consistent with the original interview guide used in the first study. Section three was newly added, in which data were not coded but used to determine what people think about COVID-19 vaccines. The detailed interview guide is displayed in Supplementary Table S1.

### Study participants

This study followed Chinese residents who participated in the interviews from February to March 2021 (16). The respondents were interviewed face-to-face, by telephone, or *via* the Internet. Individual face-to-face, in-depth interviews were preferred. Restricted by local epidemic prevention and control measures or cross-region population mobility, face-to-face video calls *via* the Internet were used. Those participants who had poor network communication were interviewed by telephone. This study was conducted from January to mid-February 2022. Based on the last interview quality assessment, cooperation with the interview, availability of revisits, and willingness to be interviewed, 60 participants from the last interview were included in this study. The participants came from 12 provinces across mainland China: Anhui, Gansu, Guangxi, Hebei, Henan, Jiangsu, Jilin, Ningxia, Qinghai, Shandong, Xinjiang, and Zhejiang. The Ethics Committee of Wuxi Center for Disease Control and Prevention (2020No10) approved this study. Informed consent was obtained before completing the interview. Each participant was informed that the responses were used for research only, and personal information was protected. They could quit whenever they had issues with the ethics of this study.

### Data collection

Semi-structured interviews were conducted to analyse vaccine hesitancy among Chinese residents and its corresponding influential factors. Before the interview, the interviewees were consulted about when and where to interview. Furthermore, the interviewer requested permission to audio-record the interviews. The interviewer remained neutral throughout the interviews.

### Quality control and data analysis

The open-ended questions were designed in advance. The perspectives of instructors and experts on disease control and prevention concerning the research topic and design were collected through pre-interviews. According to feedback, the interview outline was further revised for formal interviews that proceeded smoothly. Researchers possessing medical literacy, communication skills and enthusiasm for disease control and prevention work are the local people in the participants' areas, pivotal to conducting the interviews smoothly and guaranteeing research accuracy (19). When collecting data, we concentrated on the oral expressions of the questions. We remained neutral to guarantee that the results were honest reflections of the participants' thoughts. The audio recordings were transcribed into text within a day after the interview. The text was later analyzed following Colaizzi's 7-step analysis method (20) and coded with the qualitative analysis software NVivo 11.0 (QSR International, Melbourne, Australia). For data entry, the interviewers cleaned and validated the data and provided a clear definition of the categorized framework. Then we coded the data based on the definition (coded twice by two independent coders); internal consistency was also checked. When there were issues, the coders would discuss them until a consensus was reached.

## Results

### Demographic characteristics of participants and classification framework

Sixty residents from 12 Chinese provinces with varying gross domestic product (GDP) levels (21) completed the interview, and 61.7% (*n* = 37) were female. The participants were categorized into four groups: healthcare workers (*n*=8), adults aged 18–59 years (*n* = 26), adults aged 60 years and above (*n* = 12), and parents of children aged 0–6 years (*n* = 14). See Table 1 for more detailed sociodemographic information about the respondents. When asked about the willingness to accept the COVID-19 vaccine booster, 93.3% answered “Yes” and believed vaccines would contain the COVID-19 pandemic. These responses verified the decline in vaccine hesitancy among participants, which aroused interest in discovering the factors behind vaccine hesitancy.

**Table 1:** Demographic characteristics of the study participants (*N* = 60).

**Demographic** **characteristics**	**Healthcare workers**	**Adults aged 18–59 years**	**Older people over 60**	**Parents of children aged 0–6 years**	**Total, *n* (%)**
**Gender**					
Male	0 (0.0)	13 (50.0)	6 (50.0)	4 (28.6)	23 (38.3)
Female	8 (100.0)	13 (50.0)	6 (50.0)	10 (71.4)	37 (61.7)
**GDP per capita of permanent residence (RMB 10,000)**					
3–5	2 (25.0)	5 (19.2)	1 (8.3)	3 (21.4)	11 (18.3)
5–8	3 (37.5)	11 (42.3)	0 (0.0)	3 (21.4)	17 (28.3)
>8	3 (37.5)	10 (38.5)	11 (91.7)	8 (57.1)	32 (53.3)
**Education level**					
Junior high school	0 (0.0)	3 (11.5)	4 (33.3)	0 (0.0)	7 (11.7)
High school graduate or equivalent	0 (0.0)	3 (11.5)	3 (25.0)	3 (21.4)	9 (15.0)
College or equivalent	8 (100.0)	18 (69.2)	5 (41.7)	10 (71.4)	41 (68.3)
Master's diploma or above	0 (0.0)	2 (7.7)	0 (0.0)	1 (7.1)	3 (5.0)
**Annual household income (RMB 10,000)**					
<5	1 (12.5)	5 (19.2)	1 (8.3)	1 (7.1)	8 (13.3)
5–10	1 (12.5)	9 (34.6)	5 (41.7)	5 (35.7)	20 (33.3)
11–15	4 (50.0)	6 (23.1)	2 (16.7)	2 (14.3)	14 (23.3)
>16	2 (25.0)	6 (23.1)	4 (33.3)	6 (42.9)	18 (30.0)
**Occupation**					
Government agencies and institutions		5 (19.2)	0 (0.0)	6 (42.9)	11 (21.2)
Business/enterprise		2 (7.7)	1 (8.3)	3 (21.4)	6 (11.5)
Production staff/worker		0 (0.0)	1 (8.3)	1 (7.1)	2 (3.8)
Full-time student		14 (53.8)	0 (0.0)	0 (0.0)	14 (26.9)
Soldier		1 (3.8)	1 (8.3)	0 (0.0)	2 (3.8)
Retired		0 (0.0)	9 (75.0)	0 (0.0)	9 (17.3)
Else		4 (15.4)	0 (0.0)	4 (28.6)	8 (15.4)
**Number of children**					
1				9 (64.3)	9 (64.3)
2				5 (35.7)	5 (35.7)
**Has the child played in last year's influenza vaccine**					
Yes				6 (42.9)	6 (42.9)
No				8 (57.1)	8 (57.1)
**Total, *n* (%)**	8 (13.3)	26 (43.3)	12 (20.0)	14 (23.3)	60 (100.0)

Based on the responses to the open-ended questions, the factors in the qualitative data were separated into three categories for subsequent analysis: background, physical, and psychological factors, each of which had a range of sub-categories under which different levels were set up, as shown in Supplementary Table S2. Supplementary Table S2 shows both the original themes and new themes. Supplementary Table S3 presents an overview of vaccine hesitancy factors among Chinese residents.

**Figure 1:**
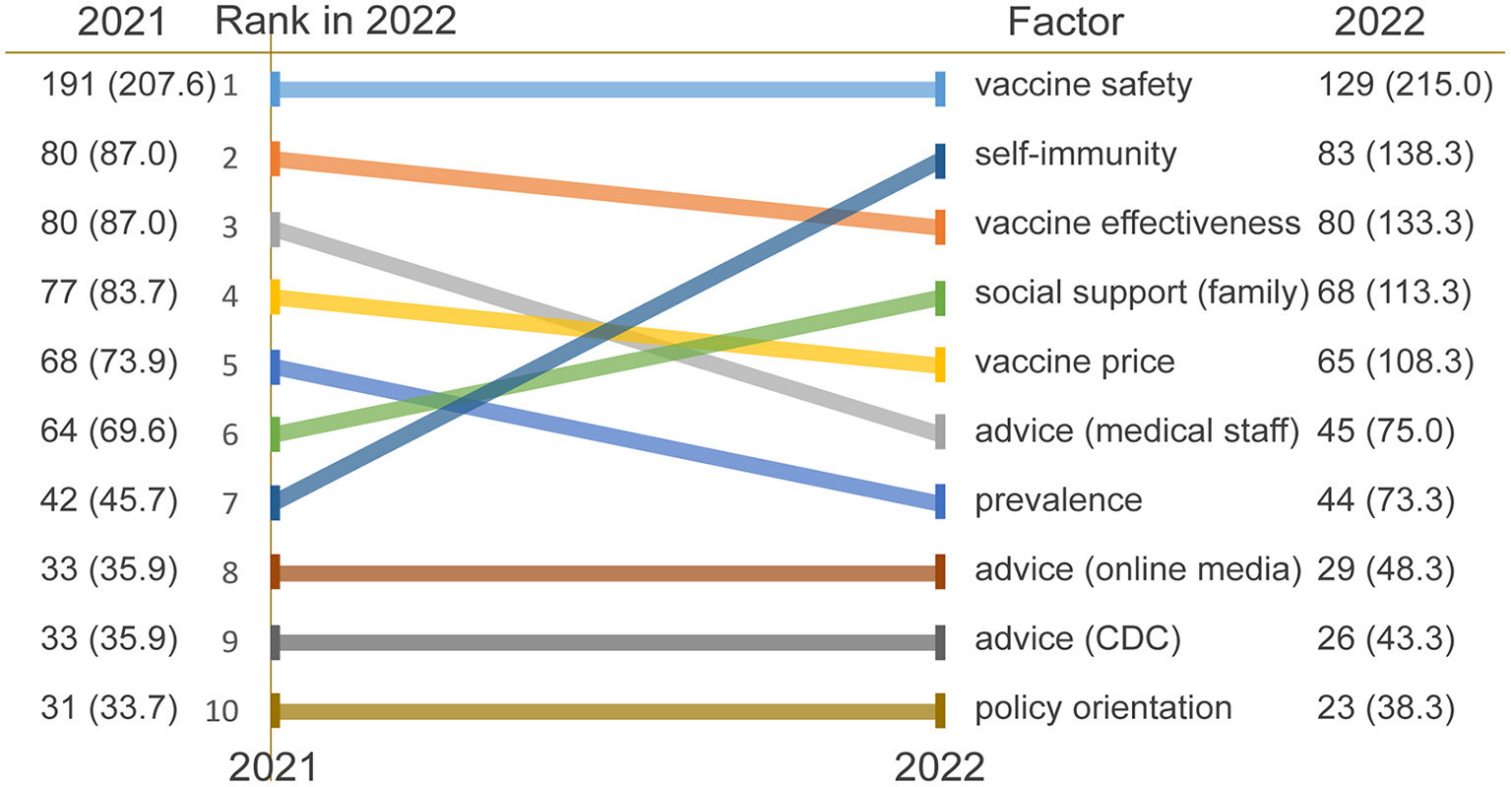
Top 10 factors influencing vaccine hesitancy in 2022. Respondents gave more than one response, so totals do not equal 100. CDC, Center for Disease Control and Prevention.

Compared with the previous study (16), the principal findings of the interviews are as follows. Vaccine safety still occupied the first-factor influencing vaccine hesitancy, followed by self-immunity, which increased by six. Vaccine effectiveness ranked third, climbing by one place. Social network support and policy orientation moved to fourth and tenth place, respectively. Noticeably, online media constituted a more substantial portion of advice sources than before, second only to medical staff. The frequencies of the top 10 factors are shown in Figure 1.

**Figure 2:**
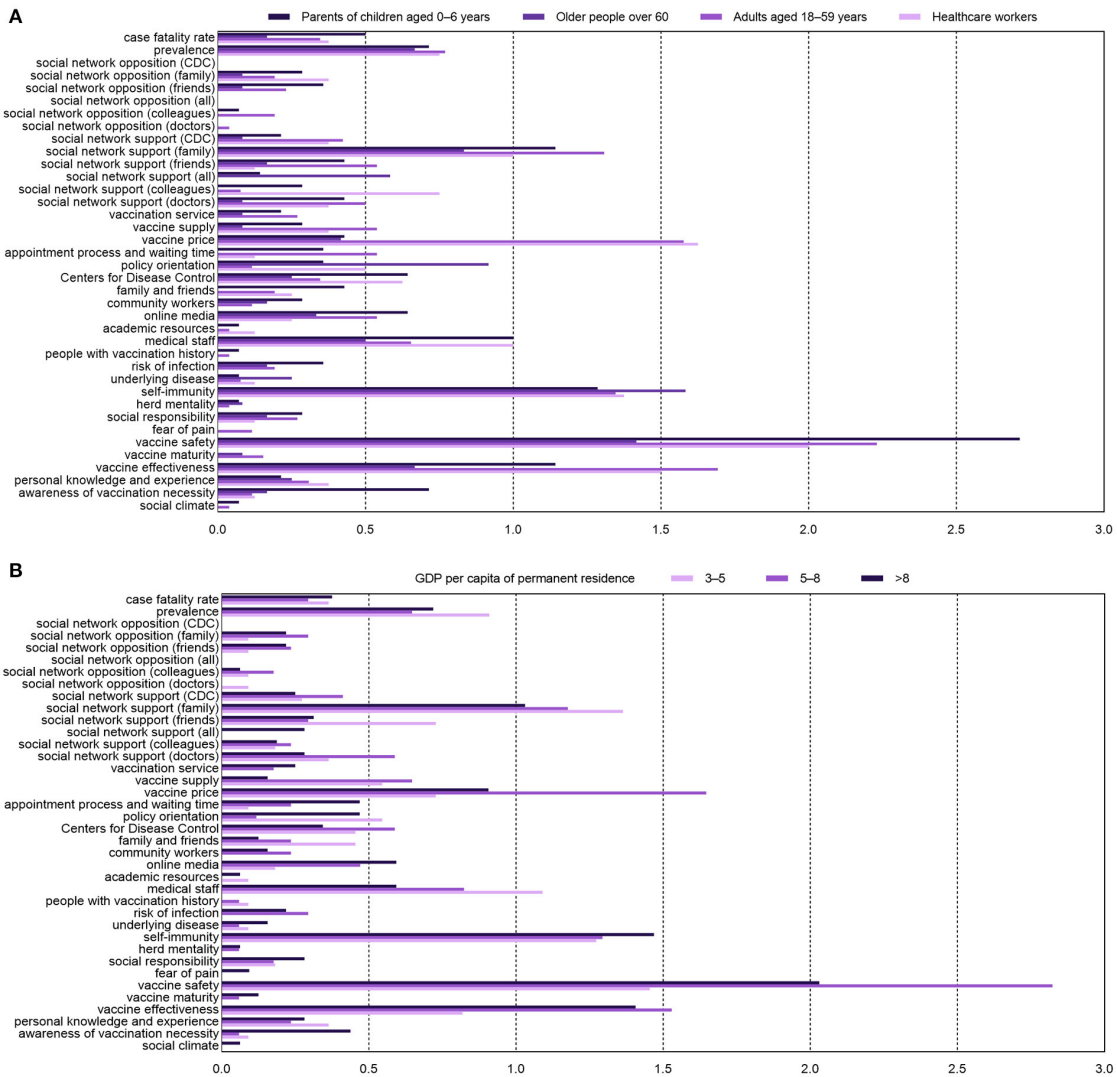
Frequency distribution of factors influencing vaccine hesitancy of four population groups **(A)** and areas with various per capita gross domestic product (GDP) of 2021 **(B)**.

The similarities and discrepancies between the four population groups are shown in Figure 2A. Medical staff and adults aged 18–59 emphasized vaccine safety, price, effectiveness, and self-immunity, which differed from the other two groups. The elderly aged 60 years and above focused on policy orientation and support from family, except for vaccine safety and effectiveness. Furthermore, support from family and advice from medical staff were two factors valued by parents of children aged 0–6 years. Moreover, those from provinces with a per capita GDP of 3–5 (RMB 10,000) were less concerned with vaccine price and effectiveness, sought advice *via* online media less, and were considerably affected by vaccination policies (Figure 2B).

### Vaccine safety

In this interview, trust in vaccine safety was the most crucial factor affecting vaccine uptake, which was aligned with the previous year's results. When asked about the most significant worries about vaccination, words such as “security,” “side effect,” and “adverse reaction” were repetitively stated, a total of 129 times. People emphasized the fear of possible adverse reactions occurring after vaccination. An adult aged 18–59 mentioned, “It is unacceptable for me to be injured due to vaccination.” Adverse reactions such as fever, chills, and swelling upset those with poor immunity and ill resistance, particularly in children and the elderly. A growing number of people have negative thoughts about the safety of biological agents for which adverse events are to blame. Worse, some feared that adverse events overpassed their effectiveness. One medical staff said, “It is essential to mention the source's reliability and safety first. It is not cost-effective to cause side effects outweighing its protective effect due to vaccination.” Except for adverse events, most residents stressed that another concern was transportation, cold-chain preservation, and contamination during shipping. By synthesizing the two interview results, vaccine safety could be a decisive factor in vaccination. People appear to refuse vaccines when they have issues with vaccine safety.

### Self-immunity

Self-immunity replacing vaccine safety has become the most influential factor influencing vaccine hesitancy in the elderly. Furthermore, self-immunity caught considerable attention in the remaining three groups. For example, self-immunity rose from seventeenth place to fourth among healthcare workers.

When asked about the need for the vaccine, terms such as “children with poor immunity,” “strengthen resistance,” and “improve immunity” were mentioned. One parent described that concern about poor immunity contributed to vaccine uptake: “Antibody vanishes entirely in 6 months after birth, leading to fragile health. Vaccination helps children develop antibodies to improve immunity and prevent diseases.” This answer was consistent with why most adults choose to be vaccinated. They desired to improve their resistance against communicable diseases, protect their health, and prevent infection *via* vaccination. Like parents, the elderly, who tend to have poorer physical fitness and weaker immunity, also emphasize fitness conditions in determining whether to vaccinate. Specifically, one elderly person expressed, “I would take pneumococcal vaccines if I am prone to pneumonia. Supposed my lungs work well, I will not take vaccination into account.” Some respondents primarily increased nutrient, fruit, and vegetable intake to enhance self-immunity during the epidemic. Those with potent immunity thought it unnecessary to receive the vaccination. One participant highlighted this, “Vaccination is not a must for those with strong immunity; for people with ill health, influenza vaccines may decrease infection risk.” The balance between self-immunity and the disease's destructive power is decisive when deciding whether to vaccinate. Vaccination may not occur if self-immunity is sufficient to cope with infectious disease hazards.

### Vaccine effectiveness

Overall, vaccine effectiveness was the third most mentioned factor influencing vaccine hesitancy. Among adults aged 18–59 years, vaccine effectiveness was second only to vaccine safety as a significant contributing factor to vaccine hesitancy, as mentioned 44 times. It is worth noting that healthcare workers and the elderly focused more on vaccine effectiveness than in the last interview.

Most participants expressed concerns about antibody titer, duration of antibody maintenance, virus mutation coverage, disease prevention effectiveness, and specific vaccine responses. Take an old man, for example, “I am worried about whether the vaccine is effective, like the COVID-19 vaccine booster. I had no idea if it could effectively prevent SARS-CoV-2 infection. Can vaccination protect against all diseases, and how long is the protection?”. Breakthrough infections aggravate vaccine hesitancy. Regarding self-funded vaccines, this concern seemed to be more evident, as another respondent depicted, “I was concerned about vaccine effectiveness. Self-funded vaccines prevent diseases that people are less likely to be infected with than free vaccines. However, the duration of antibody protection from infection is unclear. Some were even infected despite advanced vaccination.” Additionally, some participants explained their unwillingness to receive influenza vaccines because of their effectiveness. For instance, “The symptoms of influenza are mild, and one can recover quickly from simple disposal, making it unnecessary to be vaccinated. Moreover, influenza viruses mutate faster than the corresponding vaccines.” Some respondents desired open access to vaccine effectiveness trial data to enhance their understanding of vaccines and ease their concerns.

### Social network support

Social network support has become an increasingly important factor that affects vaccine hesitancy. The family dominated vaccination decisions among social support sources (family, colleagues, friends, healthcare workers, and Center for Disease Control and Prevention staff). One interviewee stated, “My family influences me significantly; I respect their advice. I interact with them daily; I will follow their opinions and get vaccinated against infectious diseases; I will even recommend that they receive vaccines.” Nevertheless, healthcare workers were less affected by family than those in the other three groups.

### Access to professional advice

Healthcare workers ranked first in both interviews in providing advice on vaccination (42.8 and 35.4 %, respectively). Social media, however, moderately undermined healthcare workers' role in providing professional suggestions, constituting a more significant proportion of advice sources (rising from 17.6 to 22.8%). When asked if they found unknown words when reading or hearing about vaccine information, 15.2% answered “often.” When asked about searching for multiple vaccine information sources, 34.8% and 41.3% chose “often” and “sometimes.” These results implied improved vaccine literacy among the participants.

### Other factors

In addition to these factors, policy orientation and the fear of pain deserve attention. Policy orientation drew more attention among the three groups than in the last interview, except for adults aged 18–59. Several parents voiced their trust in the national government policies, “I will cooperate with the national policies to inject vaccines; it seems to be more effective if the government declares, and I am willing to accept vaccination.” “Now that our country produces it, you should believe in government, so there is nothing to be concerned about.” Fear of pain was a novel factor mentioned by the participants. When talking about the barriers to vaccination, one respondent replied, “I hate injections, some people said that vaccinations caused swelling, pain, and fever, and because of this, I am hesitant.” Another participant stated, “I am afraid of pain, but it is the only way to get vaccinated.” The risk of infection gradually faded out of participants' focus, dropping from the eleventh to twenty-second, which might be closely associated with lifestyles benefiting from pandemic control. Since the COVID-19 outbreak, people have changed their lifestyles to reduce infection risk. Wearing a mask when going out, washing hands frequently, avoiding densely populated areas, replacing public transport with private cars or walking, working from home, online learning, and reducing outdoor exercise are lifestyle changes that have lowered the risk of SARS-CoV-2 infection.

## Discussion

This study conducted a return visit interview among Chinese residents to explore further the factors that affect vaccine hesitancy. The four main factors influencing Chinese residents' vaccine hesitancy were vaccine safety, self-immunity, vaccine effectiveness, and family support. Considering these above changes fully, proposing advice and possible countermeasures will help improve vaccine literacy and reduce vaccine hesitancy.

Vaccine safety and effectiveness have worried participants greatly since last year. A cross-sectional study showed that more effective and safer vaccines improved vaccination rates (22). Notably, as the controversy over these vaccines' infrequent but severe side effects grows, people cast doubt and hesitation. For instance, the human papillomavirus (HPV) vaccination rate sharply dropped from 70 to 0.6% in Japan due to misinformation on adverse events caused by the HPV vaccine (23). Therefore, increasing transparency in vaccine production, transport, supply procedures and management regarding vaccine safety is vital to dispelling doubts concerning vaccination (24). Strengthening vaccine development and production supervision, and monitoring and compensating for adverse effects following immunization are the leading measures ensuring vaccine safety and effectiveness to alleviate vaccine hesitancy (25, 26).

Despite a slight decline, vaccine price was still an obstruction in vaccination. Reimbursement for the expense of vaccines has laid the foundation for improved vaccination rates in China, similar to many other countries such as Austria, Italy, Germany, and France (27–30). Respondents were more likely to be vaccinated when vaccines were free or subsidized part of the cost. Unaffordable prices contribute to higher vaccine hesitancy (31). Decreasing cost by including it in health insurance or offering free vaccines to high-risk groups is a good way to reduce vaccine hesitancy.

The primary source of professional advice was still the medical staff. Meanwhile, advice from online media exerted a more substantial impact on vaccination than before. More recommendations from doctors boost vaccine confidence dramatically (32). So, we can strengthen the role of medical staff in facilitating vaccination. Consolidating the relevant professional knowledge of doctors through training enables them to discuss vaccines, build trust with patients and colleagues, and ultimately motivate them to accept vaccines (33). Online media is a rapid, cheap method to retrieve information. It could provide up-to-date vaccine information people need and make it possible for advisory groups to develop consultancy services *via* remote means. Even for respondents who fear pain due to vaccination, seeking help from psychological counseling through online media is conducive to reducing vaccine hesitancy (34, 35). A study demonstrated that regular exposure to vaccination messages *via* mass media contributed to vaccination (36). This inspires governments and public health agencies to disseminate real-time vaccine messages and policies through online media platforms.

Support from family has become a main focus of attention in this interview, producing more positive effects on vaccine uptake than other support. As the most basic and frequently contacted unit in a personal social network, family is closely related to obtaining emotional support, information, opinions, and knowledge. Respondents considered family members' perceptions when determining vaccination, and trusted family members significantly affected individual decisions. Accordingly, it is critical to implement comprehensive interventions for family members, including education, training courses, and post-vaccination incentives (37).

Self-immunity was the most crucial physical deciding factor, soaring to second place. A positive association between intention to be vaccinated and perceptions of becoming infected was found (38). As people learn more about COVID-19 (39), they are increasingly concerned about disease prevalence and whether self-immunity can resist the risk of infection. Vaccine recipients believed vaccines were necessary to enhance self-immunity. So, deepening people's understanding of diseases and the need to be vaccinated for self-immunity can reduce vaccine hesitancy.

This study has several limitations worthy of note. First, the participants did not represent the general population for purposive sampling. More than 88.3% of the participants had a high school diploma or higher, with more favorable opinions than others. Second, our interviews were conducted from January to mid-February 2022, just before the new immunization programmes were declared on 19 February, which might affect attitudes toward vaccination. Third, although we identified altered factors associated with vaccine hesitancy in China, this study did not elucidate the mechanism underlying these changes.

## Conclusion

We qualitatively identified changes and novel factors affecting vaccine hesitancy among Chinese residents. Our findings should remind public health authorities of evidence-based interventions to tackle vaccine hesitancy and provide policymakers with reference to successful booster vaccination to contain the COVID-19 pandemic.

## Data availability statement

The raw data supporting the findings of this study are available within the article/Supplementary material; further inquiries can be directed to the corresponding authors.

## Ethics statement

The studies involving human participants were reviewed and approved by the Ethics Committee of Wuxi Center for Disease Control and Prevention (2020No10). The patients/participants provided their written informed consent to participate in this study.

## Author contributions

Conceptualization: SL, QN, and HJ. Methodology and software: HJ. Validation: HJ and QN. Formal analysis and writing—original draft preparation: SL, JWu, SW, YZ, and HJ. Interview: SL, JWu, SW, YZ, JWa, SZ, and QN. Writing—review and editing: SL and HJ. All authors have read and agreed to the published version of the manuscript.

## Funding

This work was supported by Wuxi City Technology Development Fund (N20191007), Postgraduate Research and Practice Innovation Program of Jiangsu Province (KYCX20_0153), Jiangsu Provincial Six Talent Peak (grant number: WSN-002), and Public Health Research Center of Jiangnan University (No. JUPH201845).

## Conflict of interest

The authors declare that the research was conducted in the absence of any commercial or financial relationships that could be construed as a potential conflict of interest.

## Publisher's note

All claims expressed in this article are solely those of the authors and do not necessarily represent those of their affiliated organizations, or those of the publisher, the editors and the reviewers. Any product that may be evaluated in this article, or claim that may be made by its manufacturer, is not guaranteed or endorsed by the publisher.
