# Supplementary material for: Changes of factors associated with vaccine hesitancy in Chinese residents: A qualitative study
Source: Front Public Health. 2022 Sep 20;10:929407. doi: 10.3389/fpubh.2022.929407 (PMC9530596; doi:10.3389/fpubh.2022.929407)
Supplement: Supplementary file 1 [file Data_Sheet_1.docx]

**Supplementary Table S1 |** Semi-structured interview guide.

| **Section 1. General information** |
| --- |
| A1. Birth date  A2. Gender  A3. Nationality  A4. Current residence  A5. Education level  A6. Occupation  A7. Working in healthcare related industries  A8. Annual household income  A9. Number of children  A10. Has the child played in last year’s influenza vaccine |
| **Section 2. Questions about Non-EPI vaccine hesitancy** |
| Q1. Do you think taking the Non-Expanded Program on Immunization (Non-EPI) vaccines is necessary?  If so, what do you think are the advantages?  Q2. If not, what do you think are the disadvantages?  Q3. What worry you most if receiving Non-EPI vaccines?  Q4. What else would you think of when wanting to get Non-EPI vaccines?  Q5. Who will support you in taking Non-EPI vaccines?  Q6. Who will oppose you to taking Non-EPI vaccines?  Q7. Who will have the most influence on your decision？  Q8. Who do you turn to for help when you do not know how to decide?  Q9. When considering taking Non-EPI vaccines, what motivates you to get vaccinated?  Q10. What causes you to be unwilling to get vaccinated?  Q11. Do you have any thoughts on vaccinations?  Q12. If you decide to or not to take Non-EPI vaccines, what lead to your final decision? |
| **Section 3. Questions about COVID-19 vaccines** |
| Q13. When reading or hearing about the information on COVID-19 vaccines:  (1) Do you find any words you do not know?  (2) Do you find it hard to understand?  (3) Do you need much time to understand it?  (4) Do you need help to understand it?  Q14. When seeking information about COVID-19 vaccines:  (1) Do you search multiple information sources?  (2) Do you find the information you are looking for?  (3) Do you have the opportunity to use the information?  (4) Have you discussed your perspective on vaccination with doctors or others?  (5) Have you considered the data collected relevant to your situation?  (6) Have you considered the credibility of the information source?  (7) Have you checked the accuracy of the information？  (8) Do you find any useful information to decide whether to get vaccinated?  Q15. What do you know about COVID-19 vaccines? Are you willing to take COVID-19 vaccines? Are you willing to take a booster shot of COVID-19 vaccines? Why? Do you believe that vaccination will control the development of the epidemic?  Q16. Relative to before the outbreak, within the context of chronic COVID-19 pandemic prevention and control, what are the changes in your lifestyles (e.g. daily routine, dieting, activities, transportation and learning)? |

**Supplementary Table S2 |** An overview of the original and new themes.

|  | | original themes | new themes |
| --- | --- | --- | --- |
| Physical  deciding factor | Physical condition | self-immunity | self-immunity |
|  |  | underlying disease | underlying disease |
|  | Risk of infection | risk of infection | risk of infection |
| Background  deciding factor | Access to professional advice | medical staff | medical staff |
|  |  | Centers for Disease Control | Centers for Disease Control |
|  |  | online media | online media |
|  |  | family and friends | family and friends |
|  |  | people with vaccination history | people with vaccination history |
|  |  |  | **academic resources** |
|  |  |  | **community workers** |
|  | Social network support | support | **doctors** |
|  |  |  | **colleagues** |
|  |  |  | **friends** |
|  |  |  | **family** |
|  |  |  | **Centers for Disease Control** |
|  |  |  | **all** |
|  | Social network opposition | opposition | **doctors** |
|  |  |  | **colleagues** |
|  |  |  | **friends** |
|  |  |  | **family** |
|  |  |  | **Centers for Disease Control** |
|  |  |  | **all** |
|  | Disease condition | prevalence | prevalence |
|  |  | case fatality rate | case fatality rate |
|  |  | cure rate | cure rate |
|  | Policy orientation | policy orientation | policy orientation |
|  | Vaccine convenience | vaccine price | vaccine price |
|  |  | vaccination service | **vaccination service** |
|  |  | geographical accessibility |  |
|  |  | vaccine supply | vaccine supply |
|  |  | appointment process and waiting time | appointment process and waiting time |
|  |  | No specific reason given |  |
| Psychological  deciding factor | Trust | vaccine safety | vaccine safety |
|  |  | vaccine maturity | vaccine maturity |
|  |  | vaccine effectiveness | vaccine effectiveness |
|  | Complacency | awareness of vaccination necessity | awareness of vaccination necessity |
|  |  | personal knowledge and experience | personal knowledge and experience |
|  |  | social climate | social climate |
|  | Herd mentality | herd mentality | herd mentality |
|  | Social responsibility | social responsibility | social responsibility |
|  | **Fear of pain** |  | **fear of pain** |

Bold indicates changes.

**Supplementary Table S3 |** An overview of factors influencing vaccine hesitancy in China.

|  | | | **Healthcare workers** | **Adults aged**  **18–59 years** | **Older people**  **over 60 years** | **Parents of children**  **aged 0–6 years** |
| --- | --- | --- | --- | --- | --- | --- |
| **Physical**  **deciding factor** | **Physical condition** | **self-immunity** | 11(13.3) | 35(42.2) | 19(22.9) | 18(21.7) |
|  |  | **underlying disease** | 1(14.3) | 2(28.6) | 3(42.9) | 1(14.3) |
|  | **Risk of infection** | **risk of infection** | 0(0.0) | 5(41.7) | 2(16.7) | 5(41.7) |
| **Background**  **deciding factor** | **Access to professional advice** | **medical staff** | 8(17.8) | 17(37.8) | 6(13.3) | 14(31.1) |
|  |  | **Centers for Disease Control** | 5(19.2) | 9(34.6) | 3(11.5) | 9(34.6) |
|  |  | **online media** | 2(6.9) | 14(48.3) | 4(13.8) | 9(31.0) |
|  |  | **family and friends** | 2(15.4) | 5(38.5) | 0(0.0) | 6(46.2) |
|  |  | **people with vaccination history** | 0(0.0) | 1(50.0) | 0(0.0) | 1(50.0) |
|  |  | **academic resources** | 1(33.3) | 1(33.3) | 0(0.0) | 1(33.3) |
|  |  | **community workers** | 0(0.0) | 3(33.3) | 2(22.2) | 4(44.4) |
|  | **Social network support** | **doctors** | 3(13.0) | 13(56.5) | 1(4.3) | 6(26.1) |
|  |  | **colleagues** | 6(50.0) | 2(16.7) | 0(0.0) | 4(33.3) |
|  |  | **friends** | 1(4.3) | 14(60.9) | 2(8.7) | 6(26.1) |
|  |  | **family** | 8(11.8) | 34(50.0) | 10(14.7) | 16(23.5) |
|  |  | **Centers for Disease Control** | 3(16.7) | 11(61.1) | 1(5.6) | 3(16.7) |
|  |  | **all** | 0(0.0) | 0(0.0) | 7(77.8) | 2(22.2) |
|  | **Social network opposition** | **doctors** | 0(0.0) | 1(100.0) | 0(0.0) | 0(0.0) |
|  |  | **colleagues** | 0(0.0) | 5(83.3) | 0(0.0) | 1(16.7) |
|  |  | **friends** | 0(0.0) | 6(50.0) | 1(8.3) | 5(41.7) |
|  |  | **family** | 3(23.1) | 5(38.5) | 1(7.7) | 4(30.8) |
|  |  | **Centers for Disease Control** | 0(0.0) | 0(0.0) | 0(0.0) | 0(0.0) |
|  |  | **all** | 0(0.0) | 0(0.0) | 0(0.0) | 0(0.0) |
|  | **Disease condition** | **prevalence** | 6(13.6) | 20(45.5) | 8(18.2) | 10(22.7) |
|  |  | **case fatality rate** | 3(14.3) | 9(42.9) | 2(9.5) | 7(33.3) |
|  |  | **cure rate** | 0(0.0) | 0(0.0) | 0(0.0) | 0(0.0) |
|  | **Policy orientation** | **policy orientation** | 4(17.4) | 3(13.0) | 11(47.8) | 5(21.7) |
|  | **Vaccine convenience** | **vaccine price** | 13(20.0) | 41(63.1) | 5(7.7) | 6(9.2) |
|  |  | **vaccination service** | 0(0.0) | 7(63.6) | 1(9.1) | 3(27.3) |
|  |  | **vaccine supply** | 3(13.6) | 14(63.6) | 1(4.5) | 4(18.2) |
|  |  | **appointment process and waiting time** | 1(5.0) | 14(70.0) | 0(0.0) | 5(25.0) |
| **Psychological**  **deciding factor** | **Trust** | **vaccine safety** | 16(12.4) | 58(45.0) | 17(13.2) | 38(29.5) |
|  |  | **vaccine maturity** | 0(0.0) | 4(80.0) | 1(20.0) | 0(0.0) |
|  |  | **vaccine effectiveness** | 12(15.0) | 44(55.0) | 8(10.0) | 16(20.0) |
|  | **Complacency** | **awareness of vaccination necessity** | 1(6.3) | 3(18.8) | 2(12.5) | 10(62.5) |
|  |  | **personal knowledge and experience** | 3(17.6) | 8(47.1) | 3(17.6) | 3(17.6) |
|  |  | **social climate** | 0(0.0) | 1(50.0) | 0(0.0) | 1(50.0) |
|  | **Herd mentality** | **herd mentality** | 0(0.0) | 1(33.3) | 1(33.3) | 1(33.3) |
|  | **Social responsibility** | **social responsibility** | 1(7.1) | 7(50.0) | 2(14.3) | 4(28.6) |
|  | **Fear of pain** | **fear of pain** | 0(0.0) | 3(100.0) | 0(0.0) | 0(0.0) |
